# Historical Sketch of Rush Medical College; from Address of Pres. Freer to Graduating Class, Feb. 17, 1874

**Published:** 1874-03

**Authors:** 


*Historical Sketch of Rush Medical College, from the Address of* PRESIDENT FREER *to the Graduating Class, February* 17, 1874.

At the close of this, the thirty-first annual session of Rush Medical College, in view of the somewhat peculiar relations in which the institution is now placed, it may not be uninteresting to take a brief glance at its history, material for which we gather partly from the introductory lecture to the eleventh session of the College, by our former colleague, the distinguished Dr. Daniel Brainard, and partly from personal recollections.

The first idea of the establishment of a Medical College in Chicago dates as far back as 1836. In the autumn of that year, Dr. Brainard, in connection with the late J. C. Goodhue, of Rockford, in this State, then a resident of this city, drew up the act of incorporation, which was at the ensuing session of the Legislature at Vandalia passed, and approved by the Governor the 2nd of March, 1837. Owing, however, to the financial revulsion that fell with blighting influence upon private and public enterprises alike, some of those who the year before had the means and disposition to aid and handsomely endow the institution, now found themselves without the means of supporting their own families. No action, therefore, took place under the charter before the summer of 1843. Early in the autumn of that year, the Faculty of the College was organized by the appointment of four Professors—Drs. Brainard, Blaney, McLean and Knapp. The session commenced the 4th of December ensuing, and continued sixteen weeks.

This was before the erection of any building, and the lectures were delivered in two small rooms on Clark street. The number of students attendant upon this course was twenty-two; but a single degree was conferred, the first graduate of the institution being William Butterfield, then and now a resident of this city.

During the summer of 1844 the building occupied until the close of the tenth session was erected upon the south-east corner of Dearborn and Indiana streets, upon a lot donated for the purpose by several public-spirited citizens of the "North Side." The cost of this structure was about $3,500, defrayed partly by loan, partly by subscriptions, and the remainder made up by the Faculty.

In 1855 this building was entirely remodeled and enlarged, so as to accommodate about 250 students, at an expense of $15,000, this expense being wholly sustained by the Faculty.

Among the noted men, some deceased and some yet living, connected with the Faculty during this period, may be mentioned Daniel Brainard, William B. Herrick, and Thomas Spencer, deceased; Austin Flint, now of Bellevue Hospital Medical College, New York, and widely known as a medical author; Graham N. Fitch, afterwards U. S. Senator from Indiana; John Evans, late Governor of Colorado and U. S. Senator elect from that incipient State; and Dr. J. V. Z. Blaney, successor to Dr. Brainard as President of the College Faculty, and now Emeritus Professor of Chemistry.

In 1867, the continued and increasing prosperity of the College demanding immediate and large increase of room and general facilities for instruction, an entirely new edifice was erected upon the vacant portion of the College lot, and the old structure was remodeled so as to be merely an appendage. It had two lecture rooms, each with a seating capacity of over 700; spacious laboratory, anatomical rooms, etc., etc., constituting it, probably, the best arranged if not the largest Medical College in this country or any other. The approximate cost of the whole improvement, exclusive of the original lot and building, was about $70,000, and was met solely by the members of the Faculty. The apparatus, museum, cabinets, furniture and fixtures in the building, although very valuable, can scarcely be estimated in money. Whatever the value of the whole, in a single night, the memorable 9th of October, 1871, it disappeared.

Soon after the completion of the new building the institution received a shock by the death of its founder, Dr. Brainard, necessitating the following changes in the Faculty: Dr. J. V. Z. Blaney succeeded to the Presidency; Dr. Moses Gunn, then Professor of Surgery in the Medical department of the University of Michigan, was called to the vacant chair of Surgery; and Dr. Edwin Powell appointed Professor of Military Surgery and Surgical Anatomy. Since that time there have been added the Chair of Clinical Medicine and Diseases of the Chest, and Diseases of the Eye and Ear, filled, the former by Dr. Joseph P. Ross, and the latter by Dr. Edwin L. Holmes. Drs. Henry M. Lyman and James H. Etheridge have been appointed respectively to the Chair of Chemistry and Pharmacy, and Materia Medica and Medical Jurisprudence, made vacant by the resignation of Drs. Blaney and Ingals.

Dr. Walter Hay has been appointed adjunct Professor of Principles and Practice of Medicine, assuming the particular department of Diseases of the Brain and Nervous System; Dr. F. L. Wadsworth, assistant to the Chair of Physiology and Microscopic Anatomy; and Dr. Charles T. Parkes has for several years past discharged the onerous and important duties of Demonstrator of Anatomy to general satisfaction.

Three days after the great fire, by the kindness of the County Commissioners, a large proportion of the class having returned, the lectures were resumed in the amphitheatre of the county hospital, and at the ensuing commencement the degree of M.D. was conferred upon seventy-seven candidates.

From that period to the present, our history is so familiar to you all, that further elaboration is quite unnecessary. Rush Medical College again possesses ample means of illustration, and presents for consideration a united, experienced and harmonious Faculty, almost unequaled clinical advantages, and prestige that throughout the whole country gives character and influence to its alumni. In 1872 the Faculty of the Spring Course of Rush Medical College was reorganized by competitive examinations, or *concourse*, by which means a full corps of competent instructors was selected and assigned to their respective Chairs. This method of *concourse* is new—an innovation—in the management of Medical Colleges in this country, but the executive Faculty of Rush are satisfied, by the results so far attained, that it will be a growing benefit to the institution and its patrons.

